# Evaluating the Possibility of Translating Technological Advances in Non-Invasive Continuous Lactate Monitoring into Critical Care

**DOI:** 10.3390/s21030879

**Published:** 2021-01-28

**Authors:** Robert D. Crapnell, Ascanio Tridente, Craig E. Banks, Nina C. Dempsey-Hibbert

**Affiliations:** 1 Faculty of Science and Engineering, Manchester Metropolitan University, Chester Street, Manchester M1 5GD, UK; R.Crapnell@mmu.ac.uk; 2 Intensive Care Unit, Whiston Hospital, St Helens and Knowsley Teaching Hospitals NHS Trust, Warrington Road, Prescot L35 5DR, UK; Ascanio.Tridente@sthk.nhs.uk

**Keywords:** lactate, critical care, hyperlactataemia, sensors, non-invasive, continuous monitoring

## Abstract

Lactate is widely measured in critically ill patients as a robust indicator of patient deterioration and response to treatment. Plasma concentrations represent a balance between lactate production and clearance. Analysis has typically been performed with the aim of detecting tissue hypoxia. However, there is a diverse range of processes unrelated to increased anaerobic metabolism that result in the accumulation of lactate, complicating clinical interpretation. Further, lactate levels can change rapidly over short spaces of time, and even subtle changes can reflect a profound change in the patient’s condition. Hence, there is a significant need for frequent lactate monitoring in critical care. Lactate monitoring is commonplace in sports performance monitoring, given the elevation of lactate during anaerobic exercise. The desire to continuously monitor lactate in athletes has led to the development of various technological approaches for non-invasive, continuous lactate measurements. This review aims firstly to reflect on the potential benefits of non-invasive continuous monitoring technology within the critical care setting. Secondly, we review the current devices used to measure lactate non-invasively outside of this setting and consider the challenges that must be overcome to allow for the translation of this technology into intensive care medicine. This review will be of interest to those developing continuous monitoring sensors, opening up a new field of research.

## 1. An Introduction to Lactate Metabolism

**Figure 1:**
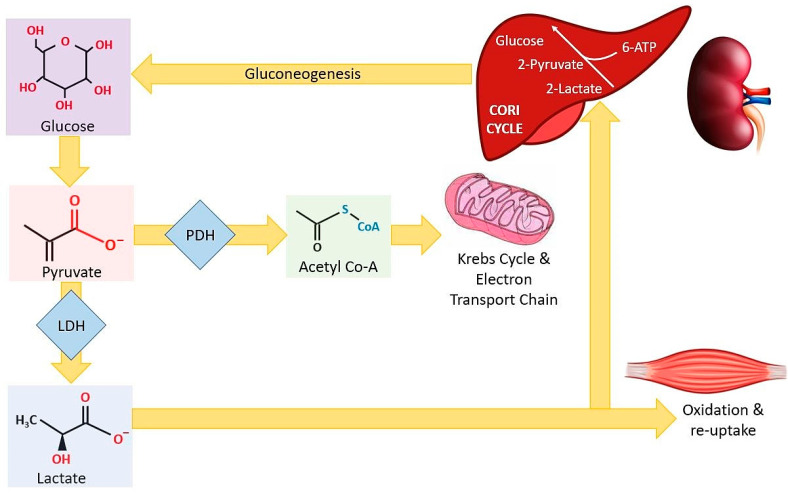
Lactate production and clearance. Under aerobic conditions, pyruvate is converted to acetyl coenzyme A (CoA) via the action of pyruvate dehydrogenase (PDH) in the cytoplasm, which enters the Krebs cycle and electron transport chain in the mitochondria. However, under anaerobic conditions, pyruvate is converted to lactate via the action of lactate dehydrogenase (LDH). Plasma lactate is cleared via action of the liver (and to a lesser degree the kidneys) though the process of gluconeogenesis. Excess lactate is oxidised and is taken up in the muscles.

Plasma concentrations of lactate represent the fine balance between lactate production and lactate clearance, and in a healthy individual, they should lie within the range of 0.5–2.2 mmol/L [1]. Lactate is ultimately produced as a result of anaerobic glycolysis, and so lactate metabolism is an integral pathway in physical exercise. Consequently, there has been huge interest and significant investment into the development of non-invasive technology for lactate measurement in professional athletes. The process of glycolysis produces an intermediate metabolite, pyruvate. Under aerobic conditions, pyruvate is converted to acetyl coenzyme A (CoA) by pyruvate dehydrogenase (PDH) to enter the Krebs cycle. However, under anaerobic conditions, pyruvate is converted by lactate dehydrogenase (LDH) to lactic acid [2] (Figure 1).

When developing technology for lactate analysis, the form of lactate within the blood is an important consideration. Lactic acid (protonated) is in equilibrium with lactate (un-protonated), its form is determined by the acid dissociation constant (pKa 3.9). At physiological pH, 7.4, it is in the form of lactate (anion). However, in the literature, the terms lactic acid and lactate are often used interchangeably. In this review, lactate will be used.

Lactate clearance is regulated by the liver, and to a lesser extent, the kidneys, via the process of gluconeogenesis (Cori cycle), in which new glucose is produced [3]. It is also now widely recognised that the presence of elevated levels of lactate within the blood (hyperlactataemia) can stimulate muscle to switch from lactate release to lactate uptake via oxidation, thereby adding a further level of complexity to the process of lactate metabolism [4,5,6]. It is also worth pointing out that lactate exists in nature in two stereoisomeric forms due to the presence of an asymmetric carbon atom. The predominant form in humans is L-lactate. This is the form routinely analysed during clinical investigations and is hence the focus of this review (simply referred to as lactate herein).

### The Importance of Lactate Monitoring in Critical Care

Hyperlactataemia is a common finding among critically ill patients, acting as a robust indicator of patient deterioration and mortality [7,8,9,10,11]. Indeed, of all laboratory parameters, lactate appears to have the strongest relationship with patient outcome in the critical care setting [12,13]. Plasma lactate analysis is relied upon to stratify patients based on the need for ongoing fluid resuscitation, risk of multiple organ dysfunction and death, and as a marker for identifying patients, requiring early aggressive resuscitation [14,15]. Therefore, the most recent lactate results are of utmost importance to the intensive care clinician when contemplating patient management. Given the importance of aerobic conditions in deciding the fate of pyruvate, the causes of hyperlactataemia have historically been subgrouped according to Cohen and Woods’ classification [16]: Type A—hyperlactataemia occurs in the presence of clinical evidence of tissue hypoxia, while Type B—hyperlactataemia (and its 3 subgroups) occurs in the absence of clinical evidence of tissue hypoxia (see Table 1). However, although this classification is often still used, it over-simplifies the complexity of lactate pathophysiology, since multiple processes are frequently present in a single patient. Therefore, it is often more useful to consider conditions in which the main mechanism for elevated lactate is either increased production or impaired clearance. Given the wide range of critical conditions in which hyperlactataemia is featured (Table 1), it is clear that lactate monitoring is fundamental on the intensive care unit (ICU). The importance of frequent/repeated measurements is evident when considering that lactate levels can change rapidly over very short spaces of time, and even subtle changes in lactate levels can reflect a profound change in the patient’s condition [17]. Indeed, dynamic lactate measurements in the ICU have been shown to be superior to static lactate measurements for predicting mortality [13,18,19,20].

One of the most important uses of lactate monitoring within the ICU is in the diagnosis and monitoring of sepsis, which is largely due to its inclusion in the Surviving Sepsis Campaign (SSC) “three-hour bundle” and subsequent “one-hour bundle”, recommending that lactate is assessed within one hour of suspicion of sepsis to guide resuscitation [21,22]. Sepsis is a life-threatening organ dysfunction due to a dysregulated host response to infection. It is the most common cause of ICU admission, and the incidence rate is increasing [23]. Interestingly, although it was initially suggested that lactate only be measured at the time of sepsis presentation, it was subsequently proposed that serial evaluation may have greater value [24,25].

Sepsis is a valid example of a condition in which the classification of the associated hyperlactataemia using the Cohen and Woods’ classification system falls short, and hence, it is even more important that lactate is monitored as frequently as possible in such patients. The hyperlactataemia in sepsis is multifactorial and certainly a consequence of both increased lactate production and impaired lactate clearance, rather than solely due to increased production from tissue hypoperfusion. Indeed, the importance of mitochondrial dysfunction [26,27], altered PDH activity [28,29], the heightened metabolic and inflammatory states [30], increased protein catabolism [31], endogenous β2 adrenergic receptor agonists [32], as well as the effects of common sepsis treatment approaches are now well recognised as causative [33]. The release of endogenous β2 adrenergic receptor agonists in some patients, due to the autonomic nervous system response to hypotension, up-regulates glycolysis, generating more pyruvate than can be used by the mitochondria. These hyperlactataemic patients are in a catecholamine-dependent shock state, which will eventually result in a profound hypotension and multi-organ failure if not identified promptly and managed appropriately. In stark contrast, the use of exogenous β2 adrenergic receptor agonists during management of the sepsis-induced hypotension results in vast clinical improvement of the patient, while lactate levels continue to rise [34]. Thus, there is evidence that an exogenous epinephrine-induced rise in lactate may be a positive indicator of effective treatment [35]. The multifactorial nature of the hyperlactataemia in this setting provides solid justification for continuous lactate monitoring.

## 2. Current Methodologies Used for Lactate Analysis in Critical Care

Typically, lactate is measured in whole anticoagulated blood samples on automated clinical chemistry analysers within hospital pathology departments. However, the importance of rapid lactate monitoring has driven the development of point-of-care (POC) devices for critical care and emergency departments. There is a large body of evidence demonstrating the impact of such testing, both from a patient outcome and cost-effectiveness perspective [36]. The POC devices currently used for such measurements are either portable bench-top analysers or smaller, hand-held devices that can include lactate as part of a multi-parameter testing platform or can be solely dedicated to lactate assessment. However, regardless of the platform, the typical blood sample is drawn through an intravenous (IV) access line, and this sampling must be repeated if discrete measurements to track lactate kinetics are desired. Hand-held devices typically require a small sample volume and, as such, are more appropriate than blood gas analysers for monitoring in neonatal and paediatric ICUs, in which blood sample volume is limited. At present, two principal methods of lactate estimation are in clinical use. The most frequently used is the lactate dehydrogenase (LDH) method, which relies on the spectrophotometric measurement of light absorption before and after the addition of LDH to the sample, reflecting the amount of NADH formed as lactate is metabolised. The alternative is the lactic acid oxidase (LOx) method employed by most POC devices. This platform uses amperometry to measure the current produced by hydrogen peroxide formation (from lactate) at a platinum anode. The most significant limitation of the latter method is the false positive rate, as a result of glycolate, which is a metabolite of ethylene glycol.

The need for repeated measurement of blood lactate across the broad spectrum of critical conditions, coupled with the increased workload involved in repeated sampling and also the potential for iatrogenic anaemia in critical care patients [37,38], warrants the introduction of non-invasive technology for continuous lactate monitoring.

## 3. Current Non-Invasive Technology for Lactate Measurements Outside of Critical Care

The development of non-invasive technology for monitoring the concentration levels of lactate outside of the critical care setting has seen a sharp rise in interest in recent years, with citations in this field showing a 10-fold increase since 2010. The shift toward minimally or non-invasive technology is driven by the desire to detect relevant biomarkers in very small volumes of biological fluids other than blood, primarily in athletes, but also in the healthcare setting [39,40].

**Figure 2:**
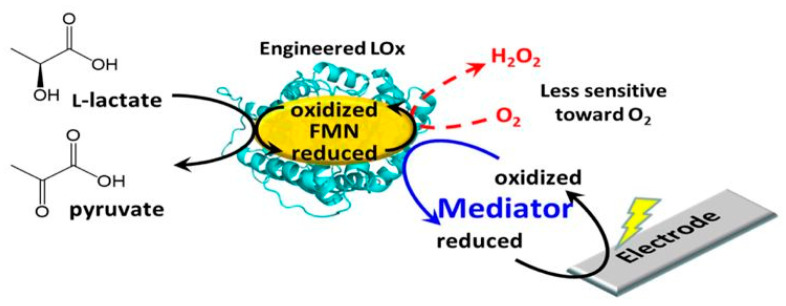
Enzymatic schematic of the engineered lactate oxidase (LOx). LOx oxidises l-lactate to pyruvate through the reduction of its co-factor, flavin mononucleotide (FMN). The engineered LOx, which is much less sensitive toward oxygen, utilises virtually only artificial electron acceptors to re-oxidise FMN. Reduced artificial electron acceptors can transfer the electrons between the LOx and the electrode. Figure reproduced from ref. [46].

The development of sensor platforms for lactate detection has typically revolved around the use of specific enzymes that react with lactate and can be tracked relatively simply; these include the commonly used LOx and LDH, along with the less prevalent lactate monooxygenase, flavocytochrome b2, and cytochrome b2 [41,42,43]. Table 2 provides an overview of the current enzymatic sensor platforms reported in the literature. LOx is the most popular enzyme found in the literature. LOx is a globular flavoprotein and works by catalysing the oxidation of lactate to pyruvate in the presence of dissolved oxygen to form hydrogen peroxide. Figure 2 provides an overview of this process. The produced hydrogen peroxide can be identified through different methodologies to predict the concentration of lactate in the sample [42,44,45].

The most common fluid matrixes used in this area of research include sweat, saliva, tears and interstitial fluid (ISF) [44,75,76,77]. It is the ability to harvest these biological fluids without piercing the epidermis that makes them so important to the future of bioanalyte sensing and monitoring. Sweat is the most promising area of non-invasive sensor technology research, with significantly more examples of sweat sensing found in the literature. This is due to its inherent advantages over the other biological fluids. ISF is difficult to access completely non-invasively using wearable platforms. Tears can be uncomfortable to extract, hard to control, and are not considered suitable for analysis outside of a research setting. Urine cannot be implemented into a wearable format, and saliva can be greatly affected by the individual’s last meal [75]. However, one of the most significant drawbacks in the use of sweat in bioanalyte sensing in general is the poor understanding of the relationship between analyte levels in sweat compared to blood and ISF [39]. Indeed, with specific regard to lactate, this is still a matter of huge debate. There are a significant number of published works reporting a strong correlation between lactate levels in sweat and in venous blood, [78,79], while there are also a number of reports that cast doubt over whether sweat lactate levels really are reflective of those in the blood, [80,81] and are more likely derived metabolically from blood glucose rather than from blood lactate. It is also important to note that lactate levels in sweat are somewhat higher than those seen in blood, with the biologically relevant lactate levels in sweat for a healthy individual ranging from 5 to 25 mM [44,75] and increasing 10-fold during physical exertion [82]. However, the general concept of sweat sensing for continuous monitoring of bioanalytes in the healthcare setting has been clinically proven with the development of sweat sensors for monitoring glucose in diabetic patients [83].

### 3.1. Sweat Lactate Sensors

**Figure 3:**
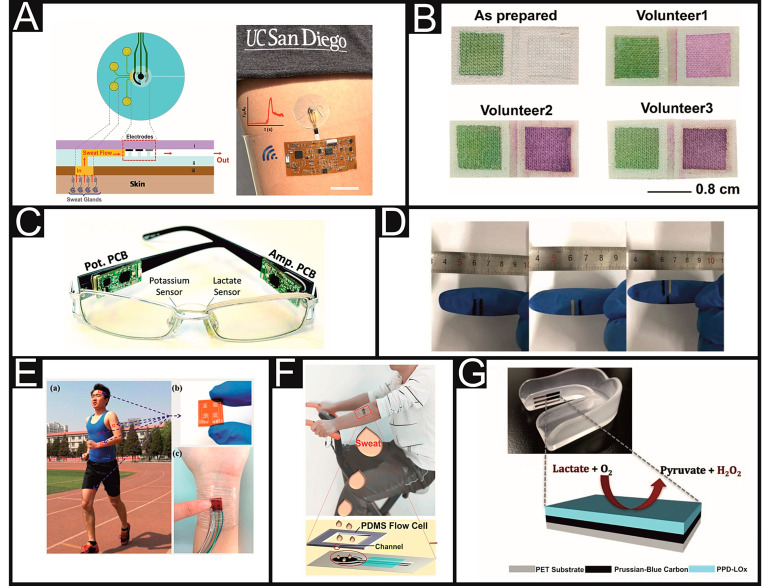
(**A**) Schematic representation and picture of the microfluidic device for the detection of lactate in sweat. Reproduced/adapted with permission from ref. [95]. Copyright 2017 American Chemical Society. (**B**) Textile-based colorimetric sensor before and after testing on three human volunteers. Reproduced/adapted with permission from ref. [96]. Copyright 2019 Elsevier. (**C**) Biosensor device integrated into glasses. Reproduced/adapted with permission from ref. [97]. Copyright 2017 Royal Society of Chemistry. (**D**) Schematic of the fabrication process for the glove-based sensor. Reproduced/adapted with permission from ref. [58]. Copyright 2018 MDPI. (**E**) (a) Electronic skin attached to the skin of a runner. (b) Optical image of the electronic skin. (c) Optical image of the electronic skin connected to a human wrist. Reproduced/adapted with permission from ref. [98]. Copyright 2017 American Chemical Society. (**F**) Screen-printed electrode (SPE) on a male volunteer’s arm while cycling. Reproduced/adapted with permission from ref. [99]. Copyright 2020 Elsevier. (**G**) Photograph and schematic of the printed biosensor on a mouthguard. Reproduced/adapted with permission from ref. [62]. Copyright 2014 Royal Society of Chemistry.

The development of suitable sensors to monitor lactate concentration in sweat has many challenges to overcome such as the direct sampling of the fluid from the skin, the collection of a large enough sample volume, transport of this sample to the recognition elements, and the accurate detection of the analyte. Additionally, these platforms must also be adaptable to become a wearable device on a living and moving subject. This can be increasingly challenging when the technology is being designed for monitoring athletic performance. As such, a large proportion of the literature focuses on the flexibility and stability of the sensors whilst using similar recognition strategies revolving around LOx with various read-out methodologies. Of all of these possible methodologies, electrochemical sensors have dominated the research field due to their accurate performance, portability, simplicity, and low-cost [84,85]. The amperometeric detection of hydrogen peroxide, a product of the LOx mechanism, in sweat, saliva and suction effusion fluid (SEF) has been reported for over 25 years [47,48,86]. This mechanistic approach is still commonplace today with research focused on various areas of improvement, such as the transition to wearables [75,87], reduction in required sample volume [46], and the modification of electrodes to improve the sensing performance [49,50]. In terms of wearable sensors, some of the main routes of application centre on patches; this includes the development of woven fabrics [51,88], screen-printed flexible and fabric patches [52,53], and tattoo mimicking-based sensor platforms [54]. Screen-printing is one of the most popular methods for electrochemical sensor fabrication due to the diversity of inks, ability to print onto a wide variety of substrates in almost any pattern, its low-cost and ease of mass manufacture [89]. As such, there have been examples reported utilising screen-printing to fabricate lactate sensors on fabric [90], tape [91], microplates [92], polyester substrates [93], and temporary tattoo transfer paper [54]. One key parameter that could be problematic for the transition of these elements to healthcare is the sample volume required. For athletic applications, this is generally not a problem due to the enhanced sweat production during physical exertion. However, in a clinical setting, where movement is significantly reduced, collecting a large enough sample volume could be problematic. This was reported in the work from Imani et al. [93], in which a wearable sensor platform was developed for the detection of lactate whilst performing a simultaneous electrocardiogram (ECG). When trialed on individuals participating in intense cycling for 15–30 min, initial readings were limited by from lack of perspiration. As the exercise period progressed, the perspiration levels increased, and excellent results were obtained. This is seen in many examples throughout the literature and highlights what could be the most important hurdle to overcome in the area of sweat sensors for clinical use [94]. However, a microfluidic system has been reported to help with the transfer of sweat directly from the glands to the 8.72 µL sample chamber by Martin et al. [95] (Figure 3A). This can fill up the chamber in 13.4 min by targeting four sweat glands that excrete sweat at 20 nL/min.

Another system employed to help with the collection of sweat has been highlighted recently by Xiao et al. [100], who used cotton pads in order to collect a sweat reservoir, which could be transported to the sensor through a silk thread that helps to guide the sample to the recognition layer. Similarly, Zhang et al. [101] used wax channels to divert the sample to its required destination. These methods were coupled to a colorimetric sensing system, which relies on the reaction of the analyte with the sensor to produce a colour change of differing intensities based on the concentration present. These paper-based colorimetric sensor platforms are a popular, simple, and inexpensive methodology in conjunction with reflectance measurements [102]. Most of the reported lactate sensors utilising colorimetric detection methodology have been focused on its implementation into textiles [55,56]. However, they do suffer from poor sensitivity and reproducibility due to an inhomogeneity of colour development, which predominantly comes from the mobility of enzymes and other reagents to the outer portions of the detection areas [103]. Promphet et al. [96] have recently reported a system (Figure 3B), with excellent coverage due to the incorporation of a surfactant stabiliser in the system. However, typically, once the system has been saturated by the sample, it will need to be replaced, making it suitable only for periodic monitoring in a clinical environment. Other examples of technology suitable for periodic monitoring include a hydrogel-based sensor that can detect lactate levels from a finger touch [57] or the implementation of a screen-printed system onto glasses (Figure 3C) [97] or disposable gloves (Figure 3D), which could be used by healthcare workers for periodic check-ups [58]. One alternative electrode production technique to screen-printing is direct current (DC) sputtering. This technique can produce ultra-thin layers of metal onto substrates to act as electrode materials.

Yokus et al. [104] utilised this methodology to produce a sensor platform to detect the lactate concentration in addition to glucose, pH, and temperature measurements attached to a watch. Improvements need to be made to extend enzymatic activity lifetimes and a reduction in the sample volume of 150 µL, if this were to transition to medical use. An improvement in the enzymatic activity and electrochemical performance has been seen by including nanomaterials such as MXene (Ti3C2Tx), due to its excellent conductivity [105]. However, this system again struggles due to sample volume, requiring a 2-min accumulation time during intense activity, which would be even longer in patients confined to a hospital bed. A large reduction in sample volume to much more appropriate levels was observed in the work by Lin et al. [59], who produced a sensor that worked over a large range (1.3–113.4 mM) utilising an ultra-low volume of 1–5 µL. This was achieved through the addition of novel sensing materials in conjunction with electrochemical impedance spectroscopy (EIS), which can measure specific changes in the surface interface caused by the lactate. However, due to the sensitivity of the sensing methodology, changes in the fluid composition will also have effects on this interface. Significant changes in the output of the sensor device when transitioning from laboratory-based tests to on-body testing are commonplace. Han et al. [98] produced a piezoelectric sensor for four analytes, lactate, glucose, uric acid and urea in sweat (Figure 3E). This “electronic skin” technology can continuously monitor in real time, using no external power supply. This drive for “self-powered” devices is increasing due to the obvious advantages of space saving and alleviating the need for battery changes or excess wiring [60,61,98,106].

As explained above, the vast majority of literature published for the non-invasive and continuous detection of lactate revolves around the use of enzymatic sensors. However, there are some examples of methodologies that do not utilise enzymes. The dependence on the use of biological recognition elements can be detrimental to the cost and life-span of developed platforms, which adds to the interest in the development of these alternative technologies, with different detection methods used, such as microwave sensors [107,108] and capacitance sensors [109]. Electrochemical detection methodologies remain the most popular in this area, with amperometeric detection mainly applied in conjunction with porous systems [110], as seen previously with glucose sensors [111,112]. Table 3 provides a summary of non-enzymatic lactate sensors.

Baba et al. [113] reported a sensor based on porous palladium film, which when used in conjunction with a Nafion membrane produced selective detection for lactate. However, these were not tested in a sweat solution and had a smaller linear range than required for a clinical setting. Poor linear range is a common theme seen for non-enzymatic lactate sensors [114]; however, Onor et al. [115] screen-printed electrodes to produce a non-enzymatic lactate sensor that displayed a far wider linear range between 1 and 180 mM. Although promising, there is little data to show interference studies and how changes in physiological matrixes would affect the system. In addition to these methods, there has been a recent surge in the use of molecularly imprinted polymers (MIPs) for sensor platforms [117]. These are polymer structures containing voids that match the size, shape, and functionalities of the target analyte. They are advantageous over their biological counterparts due to their improved thermal and chemical stabilities, low cost of production, and no exploitation of animals in their production [118]. There are limited examples of MIPs produced for the detection of lactate, but of the few, electrochemical sensing is utilised for the detection method [119]. MIPs and electrochemical detection synergise well together, as the MIPs can be formed through electropolymerisation directly onto the surface of the electrode [120]. Zaryanov et al. were one of the first to report the use of this methodology for the detection of lactate in human sweat. The sensor platform produced a wide linear range of 3–100 mM. The long sensor lifetime (>6 months) is a product of the increased stability of the polymers in comparison to enzymes. However, notoriously, the selectivity of these systems is not as good, which is due to the non-specific binding of other analytes in the systems. This work also required a larger sample volume of 50–100 µL, which would be difficult to translate to a static patient in a clinical setting. The stability of this type of sensor is highlighted by Zhang et al. [99] (Figure 3F), where their platform maintained stability and sensitivity for 7 months of storage at room temperature and after enforced bending and twisting. This system presented an even wider linear range from 1 µM to 100 mM and showed no significant interference from common constituents present in sweat.

Many of these technologies highlighted show promise but need more development in certain areas to make them suitable for using in a clinical setting. The majority will struggle from the ability to harvest enough sample volume to achieve accurate results, whereas others struggle in terms of their response to changes in the composition of the fluid (dielectric strength, pH and conductivity etc.). One area of work that could solve issues with regard to sample volume is the detection of lactate in saliva, which can be much more plentiful or stimulated without involving vigorous movement of a patient.

### 3.2. Alternative Fluid Lactate Sensors

There is far less reporting on sensor platforms for the other non-invasive biological fluids in the literature. Most likely, this is due to the difficulties in sampling or the challenges in composition changes mentioned previously; this is clearly reflected in the limited number of academic papers. The composition and properties of saliva can greatly vary depending on the meal that a person has eaten [75]. However, in a clinical environment, where food intake and timings can be controlled, this could be worked around. Again, screen-printing is used as a method of sensor fabrication due to its suitable characteristics and mass producibility. Kim et al. [62] attached a screen-printed lactate sensor to the inside of a mouthguard (Figure 3G) to produce a sensor suitable for sporting application. This uses very similar technology to those described above, which is based on LOx, but it has been tested in human saliva. It follows that this technology could be incorporated into other objects more suitable to clinical use than a mouthguard. However, there was little interference testing, and ongoing long-term stability studies were still in progress. As an alternative to screen-printing, Malon et al. [52] used a conductive paste in conjunction with moulds to form the shape of their electrodes onto cotton fabric. They also present this on the surface of a disposable glove, exhibiting the adaptability of the technology. However, all testing in saliva was done using very specific conditions and protocols, leading to questions about whether this sort of procedure is suitable to use in a real clinical environment. Mengarda et al. [116] utilised a simple potentiometric methodology to detect lactate selectively in tear samples. This sensor showed good selectivity and sensitivity, achieving a linear range between 0.1 and 10 mM, and it showed good correlation with measured blood lactate levels. However, this biological fluid is not appropriate for use in the clinical setting.

## 4. Adaption and Application of Non-Invasive Technologies into the Clinical Setting

Lactate analysis in patients admitted to the ICU is essential both for diagnostic and prognostic purposes, with lactate acting as a robust indicator of patient deterioration and also response to specific treatment strategies. It has become increasing clear that assessment of lactate kinetics provides far more useful information than a single lactate measurement taken upon ICU admission. Indeed, serial measurements have been shown to correlate well with patient prognosis and response to therapy in patients with sepsis, cardiogenic shock, respiratory failure, and mesenteric ischaemia, following post-cardiac surgery and following trauma [13,121,122,123,124]. This frequency of repeated blood sampling is not feasible in such critically ill patients, and so, there is an urgent need for the introduction of technology for non-invasive and continuous monitoring of lactate in ICU patients. Following a review of the advancements made towards biomarker sensor development in sports medicine, it is clear that the underlying technology already exists and has clear potential for translation into the healthcare setting. Much of the technology already developed is highly sensitive, with many examples of sensors detecting lactate levels in bodily fluids of <0.1 mM (Table 2). Dynamic range is also not an issue with many of the sensors in the literature being able to detect ranges of 0.1–25 mM, and some detecting lactate levels up to a maximum of 100 mM (Table 2). These sensors are capable of rapid reporting from very small sample volumes, in some cases as low as 1 µL [98], which is ideal for use in critical care settings. Furthermore, there has already been numerous successful attempts at the development of multi-analyte sensor platforms for the detection of lactate in conjunction with other useful analytes such as blood pH, glucose, and Na^+^ (Table 4), and this would be extremely useful for patient monitoring in the ICU.

However, despite the successful application of this technology within sports medicine, several important questions must be answered before such technology can be confidently introduced into routine clinical practice. Firstly, there needs to be unequivocal evidence that changes in lactate levels within the blood are accurately reflected in other bodily fluids. This indisputable evidence does not yet exist.

Research studies focusing on the correlation of lactate levels in blood with interstitial fluid [130] and subcutaneous adipose tissue [131] have reported that these fluids cannot be used as a reliable substitute for blood when monitoring lactate. Worryingly, there are reports that the lactate levels in sweat can be influenced by the site at which the sweat is collected [132], and opinion remains divided on whether sweat lactate should simply be regarded as a normal metabolic product of the sweat gland. To date, all research attempting to study the relationship between blood and sweat lactate levels has focussed on healthy individuals undergoing controlled bouts of exercise. It is not clear whether the findings from research into sweat composition of athletes can be extrapolated to that of patients in ICU suffering from conditions such as sepsis, liver failure, or respiratory failure. Secondly, it is important to determine whether the underlying stimulator of the sweating affects sweat composition. Again, the large majority of research has focussed on exercise-induced sweating. A very small number of studies have attempted to compare sweat composition following thermal and exercise stress [133] and have reported no significant differences in lactate levels, but this requires confirmation. Finally, it is vital to determine how quickly any systemic changes in lactate are reflected in the sweat or other body fluids. As stated previously, lactate levels in the blood can change rapidly over very short spaces of time in critically ill patients, and subtle changes can reflect a profound change in a patient’s condition. It is vital that these subtle changes are also seen almost simultaneously within other body fluids. The kinetics of lactate in sweat/interstitial fluid/subcutaneous adipose tissue in the critically ill needs clarification before this type of sensing can be considered further.

## 5. Conclusions

In conclusion, advancements towards the non-invasive, continuous monitoring of lactate in athletes provide real promise for the translation of this technology into critical care. This review has highlighted that the existing underlying technology for sweat sensing is adequately sensitive, stable, adaptable, and capable of analysis within minute volumes of body fluid, providing rapid results. Further research in this area would look to maximise the enzymatic activity lifetimes of the enzyme-based sensors. However, despite the existence of this technology, several key questions remain: (1) Do plasma lactate and lactate levels in secretions correlate? (2) Is the composition of exercise- and thermally-induced sweat the same? (3) Can immobile, bed-ridden patients produce enough sweat for lactate analysis? (4) Is sweat composition consistent across different sites of the body? If definitive answers to these questions can be realised, this novel technology is readily available for translation and has huge potential to change current practices in the management of critical care patients.

## Figures and Tables

**Table 1:** Cohen and Woods’ classification of hyperlactataemia; Type A occurs in the presence of tissue hypoxia, while Type B occurs in the absence of tissue hypoxia. Key evidence demonstrating the association of hyperlactataemia with patient outcome in each setting is highlighted, providing justification for serial lactate monitoring.

Cohen & Woods’ Classification	Cause	Clinical Conditions in Which the Hyperlactaemia Is Observed
A	Global Hypoxia	Shock (Cardiogenic, obstructive, distributive, hypovolaemic), sepsis, profound hypotension, severe anaemia, cardiac arrest, trauma.
	Regional hypoxia	Mesenteric ischaemia, limb ischaemia, localised trauma, microcirculatory dysfunction.
	Exertional hypoxia	Seizures, acute asthma or other increased work of breathing.
B1	Lactate elevation associated with underlying disease processes	Malignancy, sepsis, liver dysfunction, renal insufficiency, diabetic ketoacidosis, alcoholic ketoacidosis.
B2	Lactate elevation caused by drugs or toxins	Metformin, acetaminophen, β2 adrenergic receptor agonists, sympathomimetics, nucleoside reverse-transcriptase inhibitors, alcohol, cyanide, carbon monoxide.
B3	Lactate elevation caused by congenital errors of metabolism	Mitochondrial myopathy Pyruvate Dehydrogenase deficiency, glucose-6 phosphatase deficiency, congenital mitochondriopathies.

**Table 2:** Summary of enzymatic sensor platforms for the detection of lactate, highlighting the enzyme, materials used, method of detection, biological medium, linear range, and limit of detection.

Enzyme	Sample Medium	Sensor Material	Method of Detection	Limit of Detection	Linear Range	Reference
LOx ^a^	Saliva	SPE ^f^/Methocel	Electrochemiluminescence	5 µM	10–500 µM	[45]
LOx ^a^	Tears	SPE ^f^	Amperometric	-	0.39–16.60 mM	[46]
LOx ^a^	Suction effusion fluid	Pt	Amperometric	-	0.5–25 mM	[47]
LOx ^a^	Sweat	Pt/PTFE ^g^	Amperometric	-	0–120 mg dL^−1^	[48]
LOx ^a^	Saliva	PB ^i^-SPE ^f^	Amperometric	0.01 mM	0.025–0.25 mM	[49]
LOx ^a^	Saliva	Graphite/Os/PPhenol	Amperometric	13 µM	0.1–1 mM	[50]
LOx ^a^	Aqueous	Nylon/carbon ink	Amperometric	-	4–20 mM	[51]
LOx ^a^	Saliva	PB ^i^-SPE ^f^	Amperometric	0.3 mM	0.1–5 mM	[52]
LOx ^a^	Aqueous	Au/TTF ^p^-CNT ^h^	Amperometric	-	0–24 mM	[53]
LOx ^a^	Sweat	SPE ^f^/CNT ^h^	Amperometric	-	1–20 mM	[54]
LOx ^a^	Aqueous	Evolon fabric	Colourimetric	-	<5 mM>	[55]
LOx ^a^	Sweat	SU-8 polymer	Colourimetric	-	0–11 mM	[56]
LOx ^a^	Sweat	PB ^i^-SPE ^f^	Potentiometric	-	0–1 mM	[57]
LOx ^a^	Sweat	CNT ^h^	Amperometric	6.0 µM	0.047–1.52 mM	[58]
LOx ^a^	Sweat	Pd/GO ^k^	EIS ^u^	1 mM	1–100 mM	[59]
LOx ^a^	Sweat	Carbon film	Potentiometric	-	0–21 mM	[60]
LOx ^a^	Sweat	PVDF ^l^/T-ZnO ^m^	Piezoelectric	-	0–8 mM	[61]
LOx ^a^	Saliva	PB ^i^-SPE ^f^/PPD^j^	Amperometric	-	0.1–1 mM	[62]
LOx ^a^	Sweat	AgNP ^c^/Nafion	Amperometric	-	1–25 mM	[63]
LOx ^a^	Saliva	PSS ^d^/PAH ^e^	Colourimetric	0.1 mM	0.6–10 mM	[64]
LOx ^a^	Sweat	Prussian Blue	OECT ^t^	-	<1 mM	[65]
LOx ^a^	Sweat	Ionogel	OECT ^t^	-	1–100 mM	[66]
LOx ^a^	Aqueous	Au/HP ^n^-ORD ^o^	OFET ^v^	66 nM	0–1 µM	[67]
LOx ^a^	Saliva/Sweat	Nitrocellulose	Chemiluminescence	0.5/0.1 mM	0–10 mM	[68]
LOx ^a^	Sweat	Au/PB ^i^	Amperometric	0.137 mM	0–5 mM	[69]
LOx ^a^	Aqueous	TPE ^q^	Fluorescence	5.5 µM	0–200 µM	[70]
LOx ^a^	Sweat	Pt	Amperometric	-	0–70 mM	[71]
LOx ^a^	Saliva/Sweat	Cu-MOF ^r^/CS ^s^/Pt/SPE ^f^	Amperometric	0.75 µM	0.00075–1 mM	[72]
LOx ^a^	Breath	PB ^i^-SPE ^f^	Amperometric	-	150 nM–1.1 mM	[73]
LDH ^b^	Sweat	SPE ^f^	Cyclic Voltammetry	10 µM	0–100 µM	[74]

^a^ lactate oxidase; ^b^ lactate dehydrogenase; ^c^ gold nanoparticle; ^d^ poly(sodium 4-styrene sulfonate); ^e^ poly(allyl amine hydrochloride); ^f^ screen-printed electrode; ^g^ poly(tetrefluoroethylene); ^h^ carbon nanotubes; ^i^ prussian blue; ^j^ poly(phenylenediamine); ^k^ graphene oxide; ^l^ poly(vinylidene fluoride); ^m^ tetrapod-shaped ZnO; ^n^ horseradish peroxidase; ^o^ osmium-redox polymer; ^p^ tetrathiafulvalene; ^q^ tetraphenylethylene; ^r^ copper metallic framework; ^s^ chitosan; ^t^ organic electrochemical transistor; ^u^ electrochemical impedance spectroscopy; ^v^ organic field-effect transistor.

**Table 3:** Summary of non-enzymatic sensor platforms for the detection of lactate, highlighting the materials used, method of detection, biological medium, linear range, and limit of detection.

Sensor Material	Sample Medium	Method of Detection	Limit of Detection	Linear Range	Reference
Cu/Nile Red	aqueous	Capacitance	-	100 nM–1 M	[109]
NiO	aqueous	Amperometric	27 µM	0.01–7.75 mM	[110]
Pd film	sweat	Amperometric	0.34 mM	0.34–15 mM	[113]
Cu/PAN ^c^/P(AN-co-AA) ^d^	artificial sweat	Resistance	-	27–270 ppm	[114]
SPCE ^a^/Fe (III)	sweat	Potentiometric	1 mM	1–180 mM	[115]
Graphite/PPy ^b^	tears, sweat, blood	Potentiometric	81 µM	0.1–10 mM	[116]

^a^ screen-printed carbon electrode; ^b^ polypyrrole; ^c^ polyacrylonitrile; ^d^ poly(acrylonitrile-co-acrylic acid).

**Table 4:** Summary of multi-analyte sensor platforms for the detection of lactate, highlighting the materials used, method of detection, biological medium, linear range, and limit of detection.

Recognition Method	Sample Medium	Additional Analytes	Sensor Material	Detection Methodology	Linear Range (mM)	Reference
LOx ^a^	Aqueous	Oxygen	Au/SWCNT ^b^/PB ^c^	Amperometric	0.05–0.85	[87]
LOx ^a^	Sweat	Glucose, ascorbic acid, uric acid, Na^+^, K^+^	SilkNCT ^g^	Amperometric	5–35	[88]
LOx ^a^	Sweat	Na^+^, NH_4_^+^	Au/TTF ^n^/CNT ^f^	Amperometric	0–30	[90]
LOx ^a^	Aqueous	Glucose	SPE ^h^/PB ^c^	Amperometric	0.48–2.59	[91]
LOx ^a^	Aqueous	Glucose	SPE ^h^/PB ^c^	Amperometric	0.083–10.4	[92]
LOx ^a^	Sweat	pH, Na^+^	SPEES ^d^/PES ^e^	Amperometric	0–28	[94]
LOx ^a^	Sweat	Glucose	SPE ^h^/PB ^c^	Amperometric	4–20	[95]
LOx ^a^	Sweat	pH	4-AAP ^i^/TOOS ^j^	Colorimetric	0–25	[96]
LOx ^a^	Sweat	Glucose	SPE ^h^/PB ^c^	Amperometric	0–14	[97]
LOx ^a^	Sweat	Glucose, uric acid, urea	ZnO nanowires	Piezoelectric	0–20	[98]
LOx ^a^	Sweat	pH	Assay Kit	Colorimetric	0–25	[100]
LOx ^a^	Sweat	Glucose, pH	SPE ^h^/*o*-PD ^o^	Colorimetric	0–0.025	[101]
LOx ^a^	Sweat	Glucose, pH, temperature	AuNP ^m^/PB ^c^	Amperometric	1–40	[104]
LOx ^a^	Sweat	Glucose, pH	CNT ^f^/Ti_3_C_2_T_x_/PB ^c^	Amperometric	0–22	[105]
LOx ^a^	Artificial Sweat	Glucose, alcohol	ZnO film	Amperometric	1–100	[125]
LOx ^a^	Aqueous	Glucose	CNT ^f^	Field Effect Transistor	pM–mM	[126]
LOx ^a^	Aqueous	Glucose	SPE ^h^	Amperometric	0.1–8	[127]
Antibody	Artificial Sweat	Cortisol	SPE ^h^/RGO ^k^	Amperometric	0.5–25	[128]
LOx ^a^	Aqueous	Glucose	CNT ^f^/NQ ^l^	Potentiometric	2.5–15	[129]

^a^ lactate oxidase; ^b^ single-walled carbon nanotubes; ^c^ prussian blue; ^d^ sulphonated polyester ether sulphone; ^e^ polyether sulphone; ^f^ carbon nanotubes; ^g^ silk fabric-derived intrinsically nitrogen doped carbon textile; ^h^ screen-printed electrode; ^i^ 4-aminoantipyrin; ^j^ N-ethyl-N-(2-hyrdoxy-3-sulfopropyl)-3-methylaniline sodium salt dehydrate; ^k^ reduced graphene oxide; ^l^ 1,4-naphthoquinone; ^m^ gold nanoparticle; ^n^ tetrathiafulvalene; ^o^ o-phenylenediamine.

## Data Availability

Not applicable.
